# Extracellular overproduction of E7 oncoprotein of Iranian human papillomavirus type 16 by genetically engineered *Lactococcus lactis*

**DOI:** 10.1186/s12896-019-0499-5

**Published:** 2019-01-24

**Authors:** Amir Hossein Mohseni, Sedigheh Taghinezhad-S, Hossein Keyvani, Vadood Razavilar

**Affiliations:** 1 Department of Microbiology, Faculty of Basic Sciences, Science and Research Branch, Islamic Azad University, Tehran, IR Iran; 2 Department of Virology, Faculty of Medicine, Iran University of Medical Sciences, Tehran, IR Iran; 3 Department of Food Hygiene, Faculty of Veterinary Sciences, Science and Research Branch, Islamic Azad University, Tehran, IR Iran

**Keywords:** *Lactococcus lactis*, HPV-16, Protein secretion, Optimization, Batch and fed-batch fermentation

## Abstract

**Background:**

We aimed at constructing *Lactococcus lactis* strains expressing HPV-16 recombinant E7 (rE7) oncoprotein and examining its overproduction ability followed by optimizing batch and fed-batch fermentations. Thereafter, in order to assess the immunogenicity of recombinant *L. lactis* cells, C57BL/6 mice were immunized by oral gavage.

**Results:**

The results suggested that recombinant strains harboring optiE7 and E7 genes produced a maximum of 4.84 and 1.91 μg/mL of rE7 in static flask experiments, while the corresponding strains gave a maximum yield of 35.49 and 14.24 μg/mL in batch experiments, respectively. Fed-batch study indicated that the concentration of rE7 protein significantly increased after feeding yeast extract plus GM17 medium. The rE7 production of the best performing strains was 2.09- and 1.48-fold higher than that of the strains during the batch fermentation. Furthermore, biomass levels were 1.98- and 1.92-fold higher than those in batch cultivation. Oral immunization of C57BL/6 mice with recombinant *L. lactis* produced significant specific IgG and IgA antibody responses in serum and vaginal fluids, respectively. Our outcomes suggest that vaccination with *L. lactis* expressing rE7 can generate significant protective effects against E7-expressing cell line. Also, our study provides evidence that the presence of large amounts of E7-specific CD4^+^ T helper and CD8^+^ T cell precursors was stimulated. Significantly higher frequencies of HPV-16 E7 specific IL-2- and IFN-γ-secreting T cells were detected in antigen-stimulated splenocytes and intestinal mucosal lymphocytes, when compared to the control groups.

**Conclusions:**

We conclude that optimization of culture conditions along with recombinant protein expression can highly stimulate both specific humoral and cell-mediated immune responses in mice after oral immunization. These promising results represent a step towards fast-tracking a vaccine against HPV-16-associated cervical cancer.

## Background

Human papillomavirus (HPV) infection represents the most important risk factor for developing cervical cancer which is the second cause of cancer-related deaths in women worldwide [1–3]. Human papillomavirus types 16 and 18 cause 60–70% of cervical cancer worldwide, with other HPV types causing virtually all the remaining cases [4]. Transformation of proteins E6 and E7 of human papillomaviruses are consistently expressed in HPV-associated cervical cancers and are required for maintenance of the transformed state [5]. Therefore, E6 and E7 could be more effective candidates as potential tumor-specific targets for T cell-based immunotherapy of cervical cancer [6, 7].

Several studies have examined the use of bacteria (such as *Salmonella* and *Mycobacterium* spp) as E7 antigen delivery vehicles to elicit an immune response against HPV-16 [8]. Unfortunately, these organisms could recover their pathogenic potential and are not totally safe for use in humans, especially in older people, children, and immunosuppressed patients [9]. However, lactic acid bacteria (LAB) are promising candidates for in vivo delivery of antigens because of their long and safe association with humans and their food. These dairy microorganisms have been used for centuries in the fermentation and preservation of numerous foods and are considered to be safe microorganisms with a GRAS (Generally Recognized As Safe) status [10, 11]. *Lactococcus lactis* (a model lactic acid bacterium) has several advantages over other well-known protein producers. For example, this bacterium does not produce LPS or any proteases, its genome has been completely sequenced, and it is easy to manipulate genetically [12]. Further, many protein expression- and targeting-systems have already been developed for *L. lactis* for the intra- or extra-cellular production of numerous proteins of viral, bacterial, or eukaryotic origins [13, 14]. In addition, oral administration with genetic engineered *L. lactis* has proved to elicit both systemic and mucosal immunity [8, 15].

To create large amounts of cells, the genetically modified bacteria or yeast are grown in large fermentation vessels containing all the nutrients they need [6, 16]. Also, in order to produce a protein of interest in fermenters, secretion is generally preferred to cytoplasmic production as it allows continuous culture and simplifies purification [17].

In the present study, we constructed an expression vector containing native and codon optimized HPV-16 E7 gene isolated from Iranian patients. Afterwards, we explored the valuable power of statistical experimental design to optimize the production of recombinant E7 during batch and fed-batch experiments. The yields of rE7 production and biomass for both strains were determined before and after optimization. To study the expression and immunogenicity of the cloned genes in recombinant *L. lactis*, C57BL/6 mice were orally immunized with the live recombinants, where fresh sera and vaginal secretion were used to determine IgG and IgA antibody titers. Also, spleen and intestinal cells were used to study the induction of antigen-specific cellular immune responses.

## Results

### In silico analysis

Various physiological and chemical properties of the target protein were assessed by ProtParam tool. These included formula: C_854_H_1372_N_222_O_268_S_12_, total number of atoms: 2728, theoretical pI: 4.78, grand average of hydropathicity (GRAVY): -0.057, total number of negatively charged residues (Asp + Glu): 26, total number of positively charged residues (Arg + Lys): 16, aliphatic index score: 95.97, and instability index (II) score: 49.97.

**Fig. 1:**
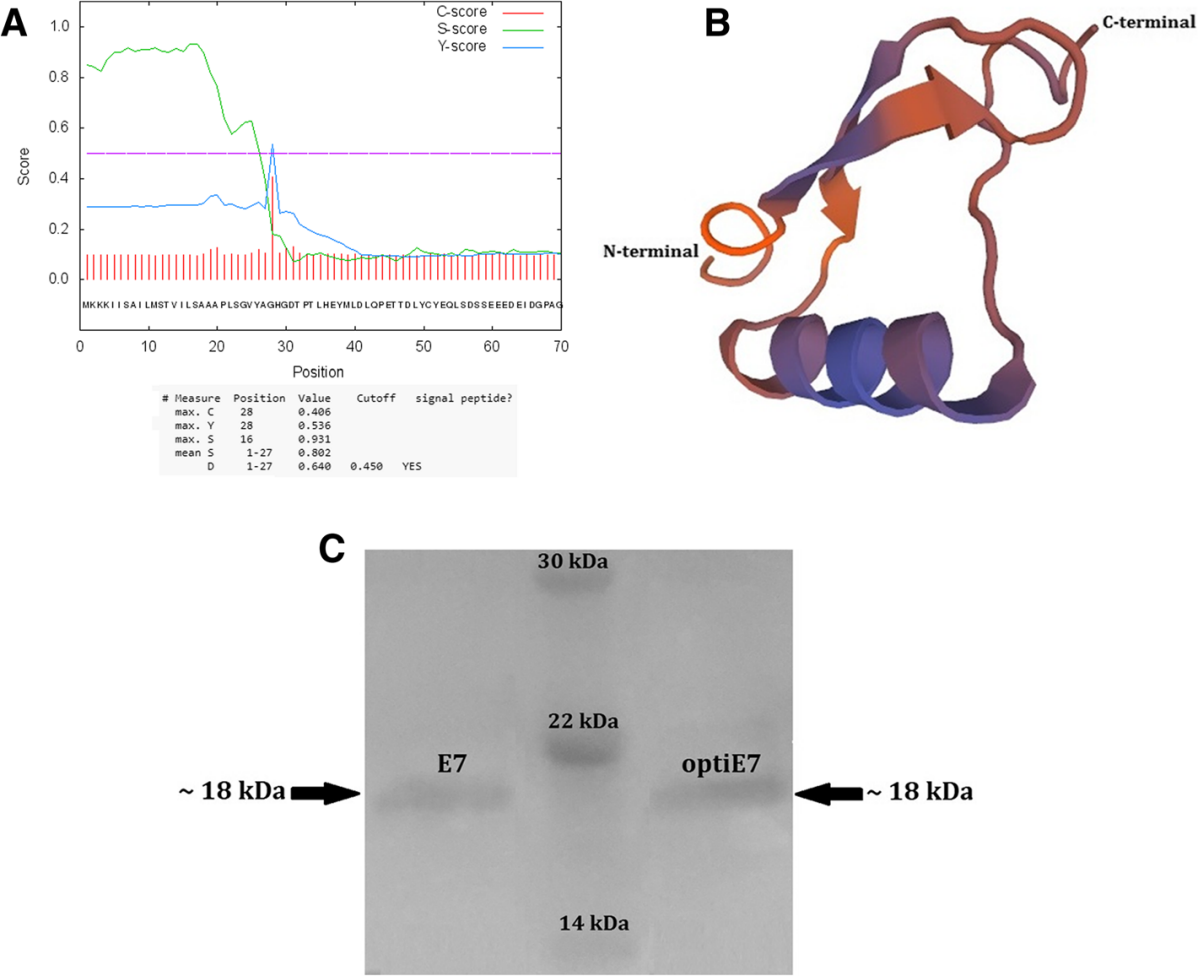
Panel **a**: The amino acid sequence of HPV-16 E7 containing SPusp45 signal peptide obtained from pNZ8123 vector was subjected to SignalP 4.1 analysis. SignalP graphical output represents the three different scores including raw cleavage site score (C-score) which is trained to distinguish signal peptide cleavage site; signal peptide score (S-score) which is trained to locate the signal peptides of a protein; and combination of C-score and the slope of S-score (Y- score). Furthermore, discrimination score (D-score) characterized the mean S and maximum Y scores, utilized for discriminating the signal peptide from its non-signal counterpart. Panel **b**: The protein tertiary structure of HPV-16 E7; the three-dimensional structure was established using SWISS-MODEL (http://swissmodel.expasy.org/). There were 13 α-helices and 10 β-sheets in the three-dimensional structure of HPV-16 E7. Panel **c**: Western blot analysis of rE7 proteins produced in *L. lactis* harboring pNZ8123-HPV16-optiE7 (right panel), prestained protein ladder (center panel), and pNZ8123-HPV16-E7 (left panel)

According to SignalP 4.1, we obtained a discrimination score (D-score) of 0.640 at the position of 27–28 amino acid residues, which was above the default cut-off point of 0.450, suggesting that there is a signal peptide at the N-terminus of recombinant HPV-16 E7 protein (Fig. 1a).

The Swiss modeling server was employed to build the tertiary structure of pNZ8123-SPUsp45-HPV16E7. The structure of C-terminal E73-K124 residues of Ld30b protein was obtained using the template PDB-2f8b (NMR structure of the C-terminal domain (dimer) of HPV-45 oncoprotein E7) as E7 protein displayed homology (in pairwise protein alignment) at NA resolution, sequence identity~ 46.00%, similarity ~ 43%, with the QMEAN score of − 1.87. The intricate spatial architecture of HPV-16 E7 indicated that there were 13 (7.38) α-helices and 10 (5.68%) β-sheets in the three-dimensional structure of pNZ8123-SPUsp45-HPV16E7 (Fig. 1b).

### Western blotting and ELISA analysis of the recombinant E7 protein

Western blot analysis was employed to test the relative level of expression of the codon optimized HPV-16 E7 and Native HPV-16 E7. Induced NZ9000 strains containing pNZ8123-HPV16-optiE7 showed an abundance of recombinant E7 protein in the supernatant fraction. The protein band migrated to about 18 kDa which is clearly visible in the Western blot analysis (Fig. 1c, right panel). The Induced NZ9000 strain containing pNZ8123-HPV16-E7 revealed a low level of the recombinant E7 protein in the supernatant, with the protein band migrating to about 18 kDa in the Western blot analysis (Fig. 1c, left panel). These results suggest that the codon optimization might synergistically improve expression of rE7 protein.

**Fig. 2:**
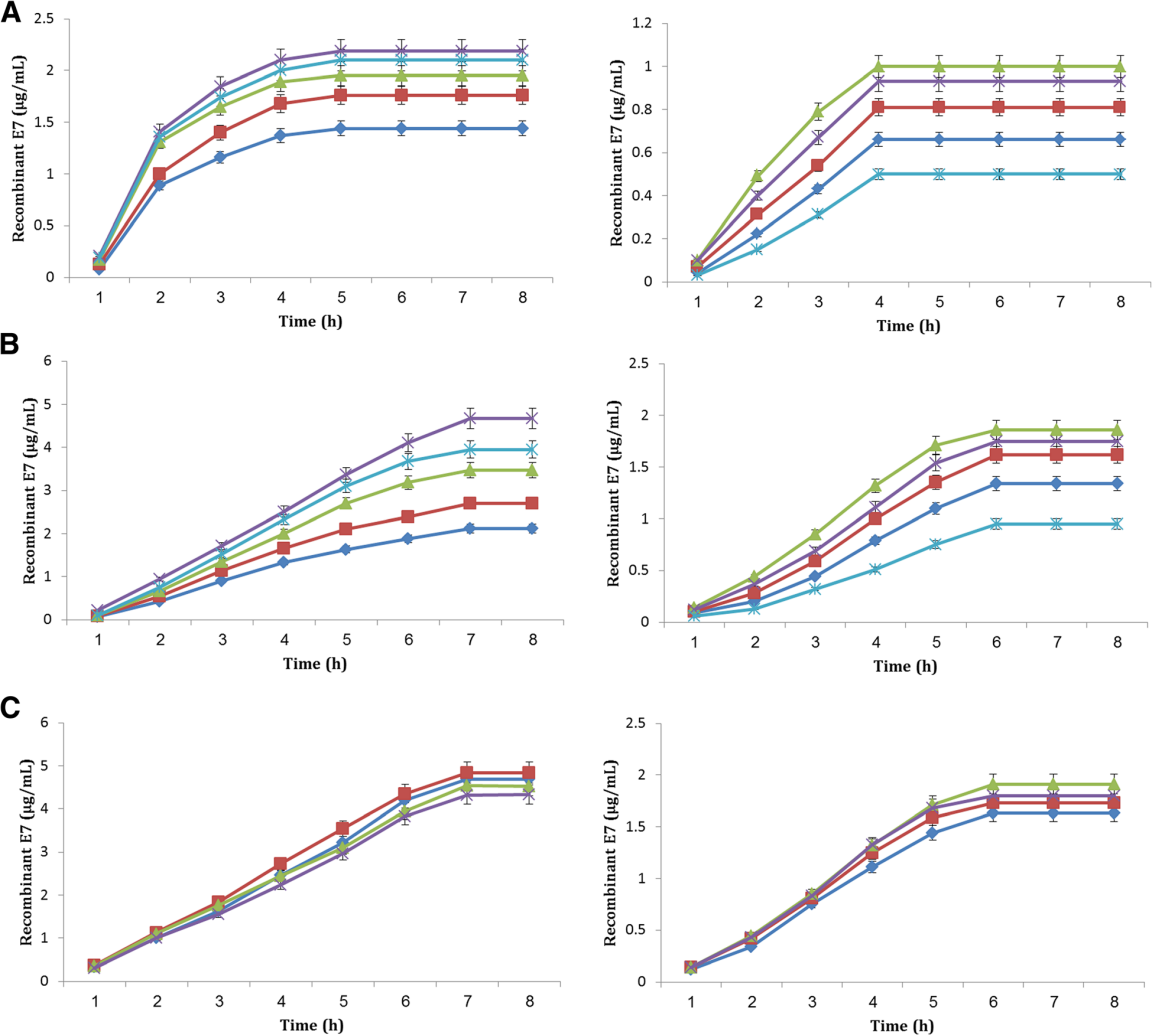
Influence of rising nisin levels and temperature 30 °C (panel **a**), growing nisin levels and temperature 20 °C (panel **b**), and induction at OD_600_ (panel **c**) at various time points on the expression of rE7 proteins in *L. lactis* containing pNZ8123-HPV16-optiE7 (Left) and pNZ8123-HPV16-E7 (right). Panel **a** and **b**: Induction time with 1 ng/mL (diamond), 5 ng/mL (square), 10 ng/mL (triangles), 15 ng/mL (multiple), and 20 ng/mL (star) nisin. Panel **c** (at 20 °C): induction at OD_600_ = 0.5 (diamond), OD_600_ = 0.6 (square), OD_600_ = 0.7 (triangle), OD_600_ = 0.8 (multiple)

Not surprisingly, the rE7 production and biomass increased steeply with elevation of nisin concentration beyond 10 (optiE7) and 5 (E7) ng/mL during the optimization process, though the optimal level was significantly different between *L. lactis* strains. Accordingly, maximum expression levels of *L. lactis* harboring optiE7 (2.19 μg/mL) and E7 (1 μg/mL) were obtained at final concentrations of 15 (optiE7) and 10 ng/mL (E7), at 30 °C, 5 and 4 h following induction remaining after 8 h (Fig. 2a).

We also found that the optimal nisin concentration coupled with lower-temperature culture conditions (from 30° to 20° C) resulted in increased ability of production of rE7 proteins in *L. lactis* harboring optiE7 (4.67 μg/mL) and E7 (1.86 μg/mL) with induction times of 7 and 6 h, respectively (Fig. 2b). However, we observed a small but consistent decline in dry weight levels in the strains.

It was observed that induction at the optical density OD_600_ = 0.6 (optiE7) and 0.7 (E7) led to high production of the recombinant rE7 protein by *L. lactis* having optiE7 (4.84 μg/mL) and E7 (1.91 μg/mL), respectively at 20 °C (Fig. 2c).

### Optimization study of batch fermentation

**Fig. 3:**
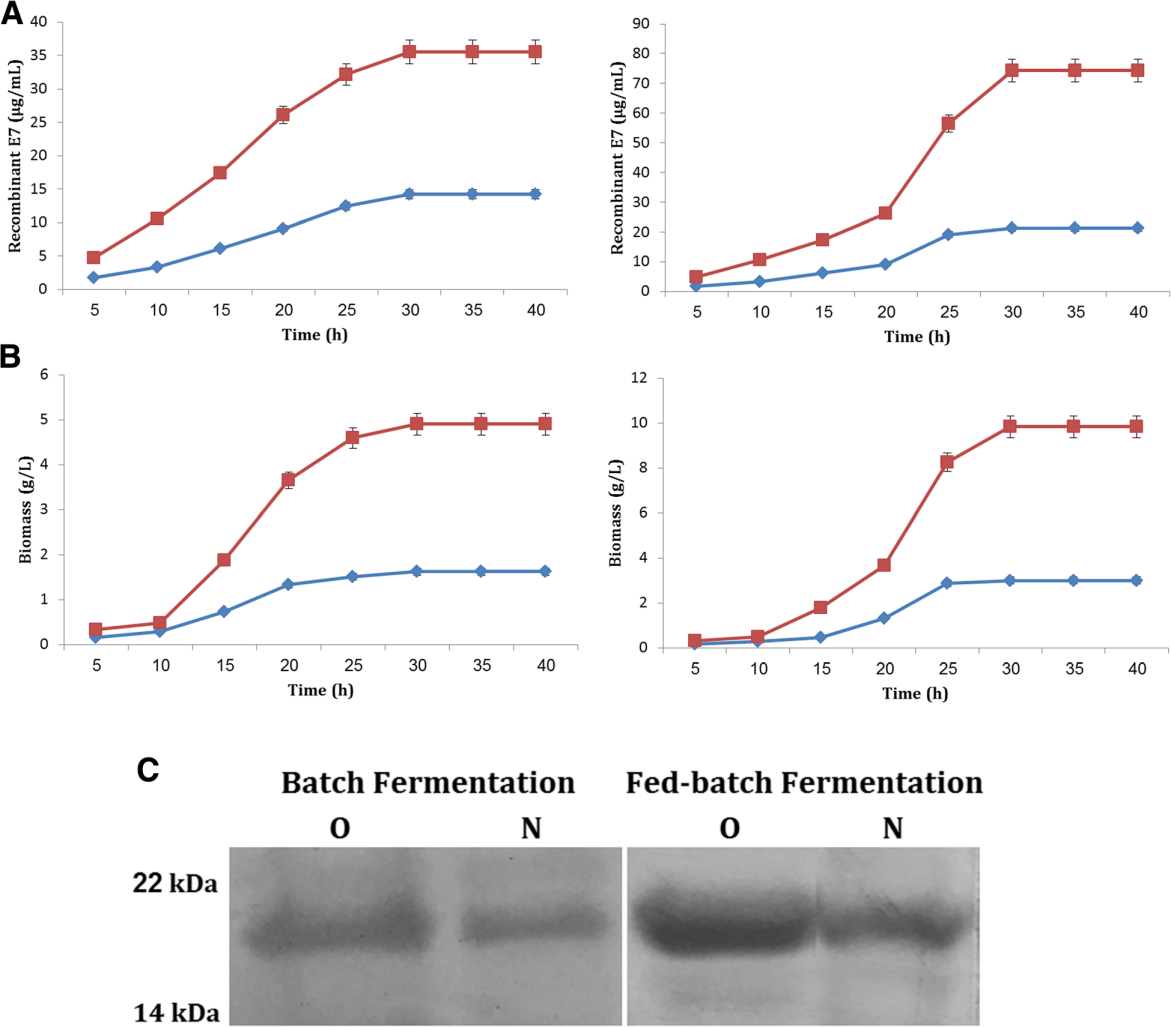
Protein expression (panel **a**) and biomass production (panel **b**), profile of *L. lactis* containing pNZ8123-HPV16-optiE7 (square) and pNZ8123-HPV16-E7 (diamond) during the course of batch (left) and fed-batch (right) fermentation; Panel **c**: Western blotting detection of rE7 proteins expressed in *L. lactis* carrying pNZ8123-HPV16-optiE7 (lane native, N) and pNZ8123-HPV16-optiE7 (lane optimized, O) during the batch (left) and fed batch (right) fermentation

In order to investigate the production of biomass and rE7 during the course of batch fermentation, both cells (*L. lactis* carrying pNZ8123-HPV16-optiE7 and pNZ8123-HPV16-E7) were induced with 15 and 10 ng/mL nisin at OD_600_ = 0.6 and 0.7, respectively. The growth was rapid, and the major part of the glucose was consumed within about 7 h after induction. The results indicated that when *L. lactis* cells were cultured in the presence of 25 g/L glucose, the cell growth, glucose utilization, and lactic acid production were significantly reduced in both strains. Similarity, the cultures grew far more slowly at high initial glucose concentrations (100 g/L), since the cells were unable to utilize all the glucose. So, rE7 expression seemed to diminish more significantly after 35–40 h. At 50 g/L of glucose, the highest biomass concentration and maximum rE7 protein yields in fermenters achieved by NZ9000 containing pNZ8123-HPV16-optiE7 were 4.95 g/L and 35.49 μg/mL at around 30 h. Also, at glucose 75 g/L, the corresponding values achieved by NZ9000 containing pNZ8123-HPV16-E7 were 1.56 g/L and 14.24 μg/mL, at around 30 h. Also, the final yields of rE7 proteins using *L. lactis* harboring pNZ8123-HPV16-optiE7 (35.2836–35.6631 with 95% CI) were close to a maximum, of 2.49 times (*P* < 0.0001) greater than those of the *L. lactis* harboring pNZ8123-HPV16-E7 vector (13.6794–14.7739 with 95% CI) (Fig. 3 (left), panel A). Further, *L. lactis* harboring pNZ8123-HPV16-optiE7 (4.7232–5.0101 with 95% CI) showed a higher biomass production (3.12-fold) compared with *L. lactis* containing pNZ8123-HPV16-E7 (1.5455–1.6945 with 95% CI) (Fig. 3 (left), panel B).

The results indicated that the maximum values of rE7 (extracellular form) concentrations were achieved by the end of the fermentation, while being still considerably higher compared to our previous batch production of intracellular rE7 oncoprotein (1.8916–2.6350 with 95% CI of difference, *P* = 0.0001 for E7 and, 1.9321–2.4746 with 95% CI of difference, *P* < 0.0001 for optiE7). Further, the maximum values for the biomass were higher than the levels reached in the previous batch study (− 0.007348–0.1540 with 95% CI of difference, *P* = 0.0651 for DCW of E7, and, − 0.1537- 0.2937 with 95% CI of difference, *P* = 0.4339 for DCW of optiE7) [12].

### Optimization study of fed-batch fermentation

The fed-batch culture was initiated following overnight batch culture with initial glucose concentrations of 50 and 75 g/L.

In the first fed-batch fermentation, the production of rE7 started at the beginning of the exponential growth phase and reached a maximum of about 68.22 and 17.83 μg/mL in this phase (for *L. lactis* containing pNZ8123-HPV16-optiE7 and pNZ8123-HPV16-E7, respectively). Accordingly, the highest maximum cell growth (8 and 2.49 g/L) was obtained after 30 h of fermentation. The maximal viable cell number was 5 × 10^9^ and 1 × 10^9^ CFU/mL, which decreased gradually upon lengthening the fermentation. Indeed, the E7 and optiE7 levels obtained at the end of this fed-batch culture were only 1.25 (3.1407–3.9193 with 95% CI of difference, *P* < 0.0001) and 1.92 (32.1766–33.5767 with 95% CI of difference, *P* < 0.0001) times higher than those obtained in the batch fermentation.

At the end of the exponential growth phase of fed-batch fermentation 2, the yields of rE7 proteins were about 74.32 and 21.19 μg/mL, respectively. The maximum biomass concentrations were 9.83 and 3 g/L, and the maximal viable cell numbers were 7.2 × 10^9^ and 3.5 × 10^9^ CFU/mL, respectively. The results obtained indicated that the feeding with yeast extract increased both the E7 and optiE7 expression by 1.18 times (range, 3.0181 to 4.4752 with 95% CI of difference, *P* = 0.0001) and 1.08 times (range, 5.0295 to 6.7771 with 95% CI of difference, *P* < 0.0001) when compared to the fed-batch fermentation 1.

In the third fed-batch experiment, the expression obtained in the exponential growth phase increased to the maximal production (70.38 and 19.55 μg/mL), and then the rE7 secretion decreased dramatically. The maximum biomass concentrations were 9.12 and 2.81 g/L when glucose was completely consumed. The maximal viable cell number was 4 × 10^9^ and 1.5 × 10^9^ CFU/mL. In this fermentation, high concentrations of glucose were able to reduce pH within a shorter time when compared to fed-batch fermentations 2. However, after 40 h of incubation, the obtained expression of E7 and optiE7 was 1.09-fold (− 8.8023–13.1123 with 95% CI of difference, *P* = 0.6560) and 1.05-fold (− 28.2592–50.2552 with 95% CI of difference, *P* = 0.5289) lower than the concentration achieved in the fed-batch fermentation 2. Overall, western blot results clearly demonstrate that rE7 production as well as biomass was significantly higher during the fed-batch culture of *L. lactis* harboring pNZ8123-HPV16-optiE7 compared to the batch studies (Fig. 3, panel C).

### Antibody, cytokine, and CTL responses following oral immunizations

**Fig. 4:**
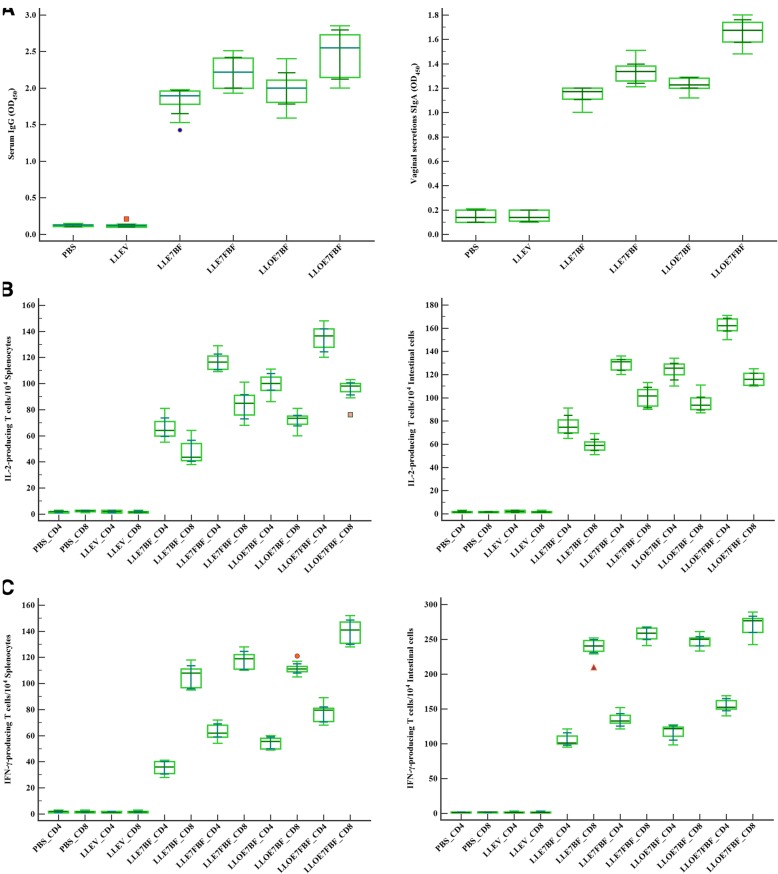
Panel **a**: The levels of E7-specific IgG and SIgA antibodies in serum (left) and vaginal discharges (right), respectively, on day 41; the sera and vaginal discharge samples were tested by indirect ELISA using recombinant human papillomavirus type 16 protein E7 as the coating antigen. Goat-anti-mouse IgG-H&L (HRP) antibody and Goat Anti-Mouse IgA alpha chain (HRP) were used as a secondary antibody. The absorbance of each microwell was read on a spectrophotometer at 450 nm. Error bars represent the mean ± standard error value of each group (*P* < 0.001). Panels **b** and **c**: Detection of E7-specific T-lymphocytes using an IL-2 (panel **b**) and IFN-γ (panel **c**) ELISPOT assay in the splenocytes (left) and intestinal mucosal lymphocytes (right); female C57BL/6 mice were immunized orally with 10^9^ CFU of *Lactococcus lactis* containing rE7 on days 1, 2, 3, 14, 15, 16, 29, 30, and 31. In order to measure cytokine production from the cells, on day 41 (ten days after the last immunization), intestinal mucosal lymphocytes and splenocytes were collected and stimulated with MHC class I - restricted peptides and MHC class II - restricted peptides (HPV-16 E7_49–57_ and HPV-16 E7_30–67_, respectively). Then, the level of E7-specific IFN-γ-producing CD8^+^ and CD4^+^ T cells and E7-specific IL-2-producing CD8^+^ and CD4^+^ T cells was calculated via mouse IFN-γ and IL-2 ELISPOT kit, respectively. The diagram demonstrates the number of spots per 10^4^ splenocytes and intestinal mucosal lymphocytes after stimulation. The results are presented as mean ± SD (n 10). PBS (Control group); LLEV: Induced *L. lactis* harboring pNZ8123; LLE7BF: Induced *L. lactis* harboring pNZ8123-HPV16-E7 produced through batch fermentation; LLE7FBF: Induced *L. lactis* harboring pNZ8123-HPV16-E7 produced through fed-batch fermentation; LLOE7BF: Induced *L. lactis* harboring pNZ8123-HPV16-optiE7 produced through batch fermentation; and LLOE7FBF: Induced *L. lactis* harboring pNZ8123-HPV16-optiE7 produced through fed-batch fermentation

To evaluate the humoral immune responses in sera, the sera were collected from all vaccinated mice on days 1, 14, 29, and 41. We examined the antibody responses in this experiment in relevant groups: PBS, LLEV, LLE7BF, LLE7FBF, LLOE7BF, and LLOE7FBF using indirect-ELISA. There was no significant serum IgG detected in the PBS and LLEV groups during the entire day (*P* = 0.7634). The production of HPV-16 E7 specific IgG in the LLE7BF, LLE7FBF, LLOE7BF, and LLOE7FBF groups was enhanced (*P* < 0.0001), suggesting that the recombinant constructs expressing rE7 activated the immune response. Nevertheless, LLE7FBF and LLOE7FBF mice groups could elicit a higher significant specific IgG titer compared to the PBS or LLEV group on days 1, 14, 29, and 41 (*P* < 0.0001) (Fig. 4 (left), panel A).

To assess whether recombinant *L. lactis* expressing rE7 could induce mucosal immune responses, vaginal washes were collected on days 1, 14, 29, 41. There was no significant SIgA produced across the groups on day 1 (*P* = 0.9372). Compared to control groups (PBS or LLEV), oral vaccination in mice could significantly elicit the SIgA antibody responses (*P* < 0.0001). On the other hand, LLE7FBF and LLOE7FBF groups induced significantly higher detectable levels of specific SIgA compared to PBS or LLEV group on days 14, 29, and 41 in the vaginal washes (*P* < 0.0001) (Fig. 4 (right), panel A).

To evaluate the amount of cytokines produced upon vaccination with recombinant *L. lactis* expressing rE7, the splenocytes and intestinal mucosal lymphocytes from immunized mice were taken and stimulated with MHC class I - restricted peptides (HPV-16 E7_49–57_) and MHC class II - restricted peptides (HPV-16 E7_30–67_). Under equal counts of *L. lactis* harboring pNZ8123-HPV16-optiE7 and pNZ8123-HPV16-E7, compared with PBS and LLEV groups, immunization of LLE7FBF and LLOE7FBF mice groups stimulated more cytokines (*P* < 0.0001), containing IFN-γ and IL-2, when compared to immunization of LLE7BF and LLOE7BF on day 41 (*P* < 0.0001) (Fig. 4b and c).

In comparison with LLE7BF_CD4 and LLOE7BF_CD4 groups, LLE7FBF_CD4 and LLOE7FBF_CD4 groups provoked nearly 1.77 time (111.9185 to 121.4815 vs. 59.9774 to 71.8226 spot-forming cells (SFC) per 10^4^ cells with 95% CI of difference, *P* < 0.0001) and 1.34 time (128.0828 to 141.3172 vs. 94.9395 to 105.8605 spot-forming cells (SFC) per 10^4^ cells with 95% CI of difference, *P* < 0.0001) as much IL-2 in splenocytes respectively (Fig. 4 (left), panel B), and nearly 1.68 time (125.3837 to 132.8163 vs. 70.5602 to 82.4398 spot-forming cells (SFC) per 10^4^ cells) and 1.31 time (157.3700 to 166.4300 vs. 117.6951 to 129.1049 spot-forming cells (SFC) per 10^4^ cells) respectively as much IL-2 in the intestinal mucosal lymphocytes (Fig. 4 (right), panel B). Also, in comparison with LLE7BF_CD8 and LLOE7BF_CD8 groups, LLE7FBF_CD8 and LLOE7FBF_CD8 groups provoked nearly 1.75 time (75.6792 to 91.1208 vs. 41.0177 to 53.7823 spot-forming cells (SFC) per 10^4^ cells with 95% CI of difference, *P* < 0.0001) and 1.32 time (89.6951 to 101.1049 vs. 67.6820 to 76.1180 spot-forming cells (SFC) per 10^4^ cells with 95% CI of difference, P < 0.0001) as much IL-2 in splenocytes respectively Fig. 4 (left), panel B), and nearly 1.71 time (95.2645 to 107.1355 vs. 55.1608 to 63.0392 spot-forming cells (SFC) per 10^4^ cells) and 1.21 time (112.7805 to 120.0195 vs. 90.1335 to 100.8665 spot-forming cells (SFC) per 10^4^ cells) respectively as much IL-2 in the intestinal mucosal lymphocytes (Fig. 4 (right), panel B). It induces the differentiation of regulatory T cells, promotes T cell growth and natural killer cell cytolytic activity, and mediates activation induced cell death.

In comparison with LLE7BF_CD4 and LLOE7BF_CD4 groups, LLE7FBF_CD4 and LLOE7FBF_CD4 groups also stimulated almost 1.78 time (59.0752 to 67.1248 vs. 32.1270 to 38.6730 spot-forming cells (SFC) per 10^4^ cells) and 1.41 time (72.7879 to 82.2121 vs. 51.9708 to 57.8292 spot-forming cells (SFC) per 10^4^ cells) more IFN-γ in splenocytes respectively (Fig. 4 (left), panel C) and almost 1.27 time (126.8711 to 140.9289 vs. 98.3404 to 111.6596 spot-forming cells (SFC) per 10^4^ cells) and 1.31 time (147.7086 to 160.8914 vs. 109.6841 to 124.9159 spot-forming cells (SFC) per 10^4^ cells) more IFN-γ in intestinal mucosal lymphocytes respectively (Fig. 4 (right), panel C). Also, in comparison with LLE7BF_CD8 and LLOE7BF_CD8 groups, LLE7FBF_CD8 and LLOE7FBF_CD8 groups also stimulated almost 1.11 time (113.3137 to 122.8863 vs. 99.9676 to 112.0324 spot-forming cells (SFC) per 10^4^ cells) and 1.25 time (133.7699 to 146.0301 vs. 108.2753 to 114.9247 spot-forming cells (SFC) per 10^4^ cells) more IFN-γ in splenocytes respectively (Fig. 4 (left), panel C) and almost 1.07 time (250.5920 to 263.6080 vs. 229.6767 to 247.5233 spot-forming cells (SFC) per 10^4^ cells) and 1.09 time (261.9172 to 282.4828 vs. 242.0938 to 253.7062 spot-forming cells (SFC) per 10^4^ cells) more IFN-γ in intestinal mucosal lymphocytes respectively (Fig. 4 (right), panel C), which is a powerful activator of macrophages, inducer of MHC I and MHC II expression, and indicator of Th1 responses.

To evaluate the E7-specific cytotoxic T lymphocyte (CTL) responses, we performed lactate dehydrogenase (LDH) activity assays using intestinal mucosal lymphocytes stimulated with 1 g/mL synthesized HPV-16 E7_49–57_ peptide which LLE7FBF. The LLOE7FBF mice group indicated a significant cellular response compared to LLE7BF and LLOE7BF groups at this ratio, with effector:TC-1 (30:1 ratio) (*P* = 0.0001). However, no significant difference was observed in the CTL activity between PBS and LLEV groups (*P* = 0.7263).

### Challenge study

In tumor protection study, compared with LLE7BF and LLOE7BF groups, LLE7FBF and LLOE7FBF groups led to a significant delay in tumor growth and an increased tumor protection on day 41 (median tumor size of 3 and 1 cm^3^, respectively) (*P* = 0.0001). In contrast, all LLEV mice groups grew tumors with a median tumor size of 6 cm^3^.

In the tumor treatment study, LLE7FBF and LLOE7FBF groups had better treatment effects on progression of tumor size in comparison with LLE7BF and LLOE7BF groups, with a median tumor size of 2.9 and 1.1 cm^3^, respectively (*P* < 0.0001). However, LLEV mice treatment groups had no significant inhibitions for tumor growth, with a median tumor size of 5.9 cm^3^.

**Fig. 5:**
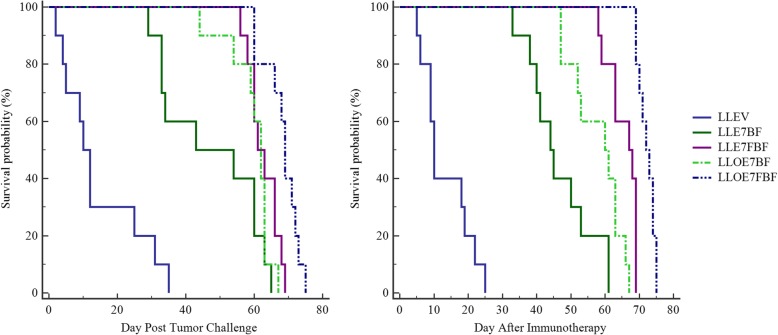
Kaplan–Meier plots of survival probability in C57BL/6-vaccinated mice (left panel) and TC-1 tumor-bearing C57BL/6 mice treated with recombinant strains (right panel); the data confirmed that immunization with *L. lactis* harboring pNZ8123-HPV16-optiE7 produced through fed-batch fermentation via the oral route generated the most powerful tumor protection (*P* < 0.0001). The survival time of tumor-bearing mice was considerably higher in the LLOE7FBF group. PBS (Control group); LLEV: Induced *L. lactis* harboring pNZ8123; LLE7BF: Induced *L. lactis* harboring pNZ8123-HPV16-E7 produced through batch fermentation; LLE7FBF: Induced *L. lactis* harboring pNZ8123-HPV16-E7 produced through fed-batch fermentation; LLOE7BF: Induced *L. lactis* harboring pNZ8123-HPV16-optiE7 produced through batch fermentation; and LLOE7FBF: Induced *L. lactis* harboring pNZ8123-HPV16-optiE7 produced through fed-batch fermentation

Finally, the median survival rate of the mice immunized with LLE7BF, LLE7FBF, LLOE7BF, and LLOE7FBF groups was 43, 61, 62, and 69% over 60, 66, 63, and 72 days after inoculation of the TC-1 tumor (*P* < 0.0001), respectively, while that of the mice immunized with LLEV group was 10% over 25 days (Fig. 5, left panel). Also, the median survival rate of the animals immunized with LLEV, LLE7BF, LLE7FBF, LLOE7BF, and LLOE7FBF group was 10, 44, 67, 60, and 72% over 19, 53, 69, 63, and 74 days respectively over 75 days after treatment (*P* < 0.0001), respectively (Fig. 5, right panel).

## Discussion

Advanced optimization can be done to attain higher expression levels with the different expression conditions. The optimization procedure was conducted given the level of nisin, codon usage optimization, cell density at induction time, and induction temperature in the growth medium to obtain a higher level of rE7 expression in *L. lactis* [12].

It was found that growth conditions in static flasks cannot be simply controlled; hence, large batch-to-batch variations may happen [6]. Production and recombinant protein expression in a fermenter allows key variables such as temperature, pH, and nutrient supply to be regulated. Considering that culture optimization is an efficient way to enhancing the overall recombinant protein yield in *L. lactis*, we further investigated the influence of culture condition in improving rE7 yields in *L. lactis* NZ9000. We observed that production in a fermenter under controlled pH conditions resulted in better glucose uptake and enhanced the productivity of the culture when compared to static flask cultures. The results obtained from the optimization study of the batch fermentation under various glucose concentrations were in accordance with our previous studies, showing higher efficiency in the production of rE7 protein and biomass in the NZ9000 strains [12].

Despite approximately 22 years of laboratory application of the NICE expression system, we did not find any publications explaining the fed-batch production of recombinant *L. lactis* NZ9000 harboring HPV-16 E7 oncogene. We also investigated the positive effect of fed-batch fermentation on overproduction of rE7 protein in both NZ9000 recombinants. In order to improve the secretion of rE7 oncoprotein, the concentrated nutrients were fed stepwise to investigate the effects of fed-batch fermentations using different feeding agents. This experimental design enabled us to discover the effect of glucose and yeast extract limitation on cell growth and rE7 production. During the course of fed-batch fermentation, biomass increased rapidly while the growth of the microorganisms entered the logarithmic phase, where the lag time of bacterial growth was shorter than that in the batch culture.

From a kinetic point of view, it can be noted that rE7 expression improved rapidly in the first period in parallel with the rapid rise in biomass and the maximum pH drop developed in the culture. On the other hand, from the ~ 7 h of fermentation, rE7 expression began to decline in combination with the reduction in both the biomass and pH drop rates. This suggested that the rE7 was produced in a pH dependent manner in this culture. It has frequently been reported that accumulation of lactic acid in the medium inhibits fermentation, resulting in lower protein levels [18]. Also, after 30 h of fermentation, recombinants of *L. lactis* producing rE7 protein lost their ability to recover the acidic condition at the end of fermentation.

The second fed-batch fermentation was performed to further improve rE7 production. Surprisingly, in the second fed-batch experiment, a profile different from the first fermentation profile was obtained. We concluded that feeding yeast extract (nitrogen source) in M17 medium supplemented with glucose reduced the carbon source consumption, thus contributing to accumulation of glucose in the medium culminating in slow reduction of pH after 20 h of incubation along with the rapid rise in the biomass and rE7 productions. This result suggested that yeast extract provides both a comparatively larger amount of free amino acids and short peptides as well as more growth factors compared to other protein hydrolysates [19–21].

These results confirmed that the biomass and rE7 concentrations obtained at the end of the first and third fed-batch culture were only slightly higher than in the batch culture using the same fermenter. It was observed that neither additional glucose supplementation (profile 1) nor glucose plus yeast extract (profile 3) significantly improved the biomass and rE7 production by *L. lactis* due to toxicity of excess lactic acid accumulated in the acidic environment. This effect was even more pronounced in the profile 1 due to its high level of lactic acid. In contrast, high levels of protein production were achieved in the second fed-batch profile. It was seen that yeast extract feeding strategy was an effective way for improving rE7 production in *L. lactis*, confirming the interactions between the nitrogen source and GM17 medium. From a practical point of view, the fed-batch culture (profile 2) revealed 1.98- and 1.92-fold rise in the cell dry weight of optiE7 and E7 compared with the batch culture (Fig. 3 (right), panel B). Also, the positive expression rates of rE7 protein significantly improved in both recombinant strains (2.09- and 1.48-fold growths) as compared with the batch experiments (Fig. 3 (right), panel A). Further, the fed-batch fermentation time was shorter than that of batch fermentation.

In this study, the ability of *L. lactis* recombinants harboring optiE7 and E7 producing rE7 during the batch and fed-batch fermentation was assessed by determining the presence of antigen-specific serum IgG and antigen-specific vaginal SIgA antibodies. Taken together, our results suggest that mice vaccinated orally with *L. lactis* expressing rE7 after prime-boost immunization can experience significant serum IgG levels and gain strong immunogenicity. It also induces significant mucosal immune responses which may contribute to protection against virus infection.

In comparison with LLE7BF and LLOE7BF groups, LLE7FBF and LLOE7FBF groups provoked more E7-specific IFN-γ-secreting CD8^+^ T cell within the spleen and intestinal cells stimulated with HPV-16 E7_49–57_ peptide (MHC class I epitope). In contrast, compared with LLE7BF and LLOE7BF groups, LLE7FBF and LLOE7FBF groups stimulated more E7-specific Il-2-secreting CD4^+^ T cells within the spleen and intestinal cells stimulated with HPV-16 E7_30–67_ peptide (MHC class II epitope). These outcomes imply that LLE7FBF and LLOE7FBF mice groups induced potent Th1 responses, which are necessary for tumor regression and considerable E7-specific antibody immune responses. Also, the results from tumor challenge revealed that oral administration of *L. lactis* expressing rE7 elicited antitumor effects against tumors in C57/BL6 mice.

Overall, expression of recombinant E7 in *L. lactis* and the observed immune response after oral immunizations are a first step towards developing an effective preparation for immunizing HPV-16-positive patients.

## Conclusions

The two major contributions of this study have been improving a suitable strategy for producing high concentrations of biomass and rE7 through batch and fed-batch fermentation and assessing the protective and therapeutic immune responses induced by recombinant *L. lactis* harboring E7 protein against HPV-16 Infection in C57BL/6 Mice. We conclude that fed-batch strategy reported in this paper can offer a significant incentive for large-scale production of recombinant E7 protein. Meanwhile, our results confirmed that both humoral and cellular immune responses could be elicited upon oral administration of recombinant *L. lactis* in C57BL/6 mice. Overall, the strategy used in this study may offer useful and cost-effective insights into developing a new generation of HPV therapeutic vaccines. However, further studies are required to assess the efficacy and safety of this recombinant construct in a double-blind, placebo controlled study of patients with cervical cancer.

## Materials and methods

### Bacterial strains, mice growth conditions, and TC-1 cell lines

The strains of *Lactococcus lactis* subsp. cremoris NZ9000 (pepN::nisRK; commonly used host of the NICE system) and *E. coli* MC1061 (an intermediate host for propagation of shuttle vector) were purchased from MoBiTec company (MoBiTec, Goettingen, Germany). Also, *E. coli* JM107 (host strain for propagation of T-vectors) was purchased from Invitrogen company (Invitrogen, Carlsbad, CA, USA). *L. lactis* NZ9000 was cultured on M17 medium (Merck, USA) containing glucose 0.5% at 30 °C. On the other hand, *E. coli* strains were cultured on Luria Bertani (LB) at 37 °C.

Specific pathogen-free (SPF) female 6- to 8-week-old C57BL/6 mice were purchased from Pasteur Institute of Iran (Tehran, IRAN). The mice were housed in appropriate conventional animal care facilities in the animal house of Iran University of Medical Sciences under standard conditions.

TC-1 cell line was donated by Prof. Hossein Keyvani. TC-1 cells were generated by co-transformation of C57BL/6 mouse lung epithelial cells with HPV-16 E6 and E7 oncogenes and the human ras oncogene. TC-1 cells were cultured in RPMI-1640 medium FCS 10%, penicillin 50 U/mL, streptomycin 50 g/mL, and G418 0.4 mg/mL incubated at 37 °C in CO_2_ 10%.

### Construction of a *L. lactis* expression vector

Native and codon-optimized DNA fragments from the HPV-16 genome spanning nucleotides 7604 to 7900 were cloned into the pTZ57R/T (TA cloning vector, ampicillin resistant) and pMD17 (T-cloning vector; lacZ; Amp^r^) vector, respectively. The coding regions of recombinant pTZ57R plasmid were PCR amplified with primers 5′-GCCGGCCATGGAGATACACCTACATT-3′ and 5′-GAGCTCTGGTTTCTGAGAACAG-3′. Also, the coding regions of recombinant pMD17 plasmid were PCR amplified with 5′- GCCGGCCATGGTGATACTCCAAC-3′ and 5′-GAGCTCTGGTTTTTGTGAACAAATTG -3′. The underlines represent the *NaeI* and *SacI* sites, respectively. Both PCR products were digested with *NaeI* and *SacI* and cloned into pNZ8123 shuttle vector (repA and repC replication elements, SPusp45 signal sequence, nisA-promoter) downstream of the secretion of signal peptide SPusp45 (signal sequence of the lactococcal major secreted protein Usp45) in-frame between the Pnis and terminator (MoBiTec, Germany) cut with the same enzyme, creating pNZ8123-HPV16-E7 and pNZ8123-HPV16-optiE7 plasmids. They were subsequently added to chemically competent *E. coli* MC1061 cells [22]. The bacteria were plated onto Luria–Bertani plates containing chloramphenicol (10 μg/mL) and streptomycin (100 μg/mL). The pNZ8123-HPV16-E7 and pNZ8123-HPV16-optiE7 vectors were transferred into Electrocompetent NZ9000 by electroporation using a Gene Pulser apparatus (Bio-Rad Laboratories, Inc., CA, USA) following the MoBiTec’s instructions (MoBiTec, Germany). GM17 agar supplemented with chloramphenicol was utilized for screening the recombinant strains. The integrity of both vectors was checked by DNA sequencing (Bioneer, Korea). Also, the expression pNZ8123 vector without insert was electro-transformed into the NZ9000 competent cells as a negative control strain.

### In silico analysis of HPV-16 E7

The physiochemical parameters of HPV-16 E7 protein were calculated by ProtParam tool of ExPASy (http://web.expasy.org/protparam/). The potential secretory signal peptide (SigP) was predicted based on SignalP v4.1 (http://www.cbs.dtu.dk/services/SignalP/) with the D-cutoff value of 0.45. In order to develop the protein tertiary structure homology modeling, 3D structure models of recombinant HPV-16 E7 sequences were developed using an automated mode of SWISS-MODEL protein structure homology modeling server (https://swissmodel.expasy.org/).

### Protein expression

Recombinant strains (NZ9000 containing pNZ8123-HPV16-optiE7 or pNZ8123-HPV16-E7) were induced by various concentrations of nisin (1, 5, 10, 15, and 20 ng/mL) at different OD_600_ densities (0.5, 0.6, 0.7, and 0.8 nm) for 8 h at various culture temperatures (20 and 30 °C). The supernatant (extracellular fraction) was collected through centrifugation (12,000 rpm for 10 min). The proteins were separated via SDS polyacrylamide gel 12% electrophoresis at 60 V for 180 min. The recombinant protein was transferred to the PVDF membrane using a semi-dry blotter (Transblot semi-dry Transfer Cell; Bio-Rad) and Western blot was performed as described previously [8, 23]. Cell Biolabs’ HPV-16 E7 Oncoprotein ELISA Kit was used for quantification of extracellular proteins according to the manufacturer’s recommendation (Cell Biolabs, Inc., USA).

### Batch and fed-batch fermentation of recombinant NZ9000 strains

Batch and fed-batch fermentation was performed in a homemade 100-L fermenter equipped with a temperature-control system, a pH meter, dissolved oxygen (DO) sensor, temperature electrode, and magnetic stirrer. The fermenter was inoculated with 50 mL of an overnight culture of each recombinant NZ9000 in M17 broth and incubated at 20 °C for 40 h with varying initial concentrations of glucose (25, 50, 75, and 100 g/L). The cultures in the log phase (OD_600_ = 0.6 ± 0.1) were induced with nisin (15 and 10 ng/mL final concentration for optiE7 and E7 respectively).

Three fed-batch experiments with various feeding profiles (after 20 h of batch culture) were examined in order to improve the protein yield and to maximize the biomass production. The feeding solutions used for the fed-batch culture contained the following formula: concentrated glucose (450 g/L) (fermentation 1), GM17 medium supplemented with yeast extract 0.5% (*w*/*v*) (fermentation 2), and mixture of glucose (450 g/L) plus GM17 medium supplemented with yeast extract 0.5% (w/v) (fermentation 3). The pH value was automatically regulated within the range of 6.5–7 by adding NaOH 4 N solution using a peristaltic pump, where a slow agitation (100 rpm) was maintained to keep the fermentation broth homogeneous. The samples were taken aseptically every 5 h at regular time intervals. The rE7 productions were compared to evaluate the process efficiency of different fermentation modes. The biomass for each sample was calculated by the culture’s optical density at 600 nm (OD_600_). The optical density was calibrated against the dry cell weight (DCW; 1 OD_600_ corresponded to 0.38 g/L DCW). The numbers of viable cell counts (CFU/mL) were determined by serial dilution and plate counts.

### Immunization of C57BL/6 mice and CTL assays

Six groups of female C57BL/6 mice (*n* = 10/group) were selected in this study. The mice group consisted of Group 1- PBS; Group 2- LLEV: Induced *L. lactis* containing pNZ8123; Group 3- LLE7BF: Induced *L. lactis* containing pNZ8123-HPV16-E7 produced through batch fermentation; Group 4- LLE7FBF: Induced *L. lactis* containing pNZ8123-HPV16-E7 produced through fed-batch fermentation; Group 5- LLOE7BF: Induced *L. lactis* containing pNZ8123-HPV16-optiE7 produced through batch fermentation; and Group 6- LLOE7FBF: Induced *L. lactis* containing pNZ8123-HPV16-optiE7 produced through fed-batch fermentation.

Four groups were immunized orally with 100 μl of PBS composed of 1 × 10^9^ CFU/mL of each induced strain expressing recombinant E7 on days 1, 2, and 3. Also, two groups (n = 10/group) were immunized orally with 100 μl of PBS where 1 × 10^9^ CFU/mL of the recombinant strain containing empty shuttle vector served as control groups. All groups also received a second immunization on days 14, 15, and 16 with 100 μl of emulsion, plus a third immunization on days 29, 30, and 31. All experimental procedures were approved by the Institutional Animal Care and Use Committee of Iran University of Medical Sciences (96–03–30-29,914).

### Indirect ELISA, ELISPOT, and CTL assays

The blood collected from the tail vein of each group of mice on days 1, 29, and 41 were centrifuged at 4000 rpm for 10 min, and the separated sera were aliquoted for detecting specific antibodies. Mice were anesthetized by an ip injection containing xylazine and ketamine (0.40 mL for 10 kg of weight, Cheminova de Mexico, SA de CV). The vaginal discharge samples were collected from each anesthetized mice with 100 μL of PBS on days 1, 29, and 41. Also, on day 41 all mice were sacrificed (under 75% CO2/25% O2 anesthesia), after which the intestinal mucosal lymphocytes and splenocytes were isolated from immunized mice according to the protocol recommended by Reißig et al. [24] and BD Bioscience, respectively.

Indirect ELISA following a standard protocol was employed to determine the specific serum IgG and secretory IgA (SIgA) in vaginal discharges. Specifically, polyvinylchloride 96-well plates were coated with recombinant human papillomavirus type 16 Protein E7 (MBS1264011; MyBioSource, San Diego, CA, USA) at 10 μg/mL in carbonate-bicarbonate buffer (pH 9.6) and incubated overnight at 4 °C. The plates were subsequently blocked, washed, and incubated individually with the two-fold diluted sera and one-fold diluted vaginal fluids for 1 h at 37 °C. They were then washed and blotted in 100 μL of goat-anti-mouse IgG-H&L (HRP) antibody (ab6789; Abcam, Canada; 1:1000 dilution) and goat Anti-Mouse IgA alpha chain (HRP) (ab97235; Abcam, Canada; 1:1000 dilution) at room temperature for 1 h.

Next, the color was created with 3,3′,5,5′,-tetramethylbenzidine (TMB) for 10 min at 37 °C. H_2_SO_4_ (2 M) (100 μl/well) was used to stop the reaction. The absorbance of each well was recorded on a spectrophotometer at OD = 450 nm**.**

To determine cytokine production from the cells, intestinal mucosal lymphocytes and splenocytes were mixed and stimulated with 1 mL of complete medium containing 10 μg/mL of major histocompatibility complex (MHC) class I - restricted peptides and major histocompatibility complex (MHC) class II - restricted peptides (HPV-16 E7_49–57_ and HPV-16 E7_30–67_, respectively). Afterwards, the number of E7-specific IFN-γ-producing CD8^+^ and CD4^+^ T cells and E7-specific IL-2-producing CD8^+^ and CD4^+^ T cells were measured using mouse IFN-γ ELISPOT kit and mouse IL-2 mouse ELISPOT kit, respectively, according to the manufacturer’s instructions (R&D Systems, Inc., Minneapolis, MN). The results were quantified by calculating the number of spot forming cells (SFC) per number of cells added to the well.

In order to determine cytotoxic activity, 10 days after the last immunization (day 41), the isolated intestinal mucosal lymphocytes were stimulated in vitro, with 1 g/mL of synthesized HPV-16 E7_49–57_ peptide in 1 mL of RPMI 1640 supplemented with FCS 10% at 37 °C under CO_2_ 5%. After 5 days, the viable intestinal mucosal lymphocytes were collected and used as effector cells, while TC-1 cells were used as target cells. The lactate dehydrogenase (LDH) activity kit (CytoTox 96® Non-Radioactive Cytotoxicity Assay, Promega, Madison, USA) was utilized to measure the effector cells against TC-1 (target cells) at ratios of 30:1 according to the manufacturer’s instructions.

### Tumor protection and tumor treatment experiments

In the tumor protection experiments, three C57BL/6 mice group (*n* = 10/group) received 10^9^ CFU recombinant *L. lactis* NZ9000 containing pNZ8123 with and without HPV-16 E7 on days 1, 2, 3, 14, 15, 16, 29, 30, and 31 via the oral route. On day 41, all mice were challenged with 10^5^ TC-1 tumor cells subcutaneously in the left rear flank.

In the tumor treatment experiments, initially, three C57BL/6 mice groups (n = 10/group) were injected (subcutaneous) with 10^4^ TC-1 tumor cells into the left rear flank. The tumor growth was measured using a caliper. When tumors were 4–6 mm in the mean diameter, all mice were orally administered as described above.

The tumors were measured with calipers and the volume of the tumor was estimated as: (length × width^2^)/2. For survival analysis, the mice were considered as dead when the tumor reached 1000 mm^3^. When the tumor size of the immunized mice exceeded 1000 mm^3^, the mice were sacrificed and dissected.

### Statistical analyses

Each independent experiment was conducted in triplicate. All data points have been represented by mean, with the 95% confidence interval (CI) of difference. The differences between means of recombinant E7 and biomass productions in batch and fed-batch fermentations and comparison of different immunization schedules were captured through analysis of variance (t-test) using the MedCalc software (version 17.6; MedCalc Software, Mariakerke, Belgium) [25]. *P*-value less than 0.01 was considered statistically significant. Also, the survival percentages were analyzed using Kaplan-Meier (KM) methods.

## Abbreviations

CI: Confidence interval
C-score: Cleavage score
CTL: Cytotoxic T lymphocyte
DCW: Dry cell weight
DO: Dissolved oxygen
D-score: Discrimination score
GRAS: Generally recognized as safe
GRAVY: Grand average of hydropathicity
HPV-16: HPV type 16
KM: Kaplan-Meier
*L. lactis*: *Lactococcus lactis*
LAB: Lactic acid bacteria
LDH: Lactate dehydrogenase
LPS: Lipopolysaccharide
MHC: Major histocompatibility complex
NICE: Nisin-controlled gene expression system
rE7: Recombinant E7
SFC: Spot-forming cells
S-score: Signal score
Y-score: Combined score
